# Iterative attack-and-defend framework for improving TCR-epitope binding prediction models

**DOI:** 10.1093/bioinformatics/btaf224

**Published:** 2025-07-15

**Authors:** Pengfei Zhang, Hao Mei, Seojin Bang, Heewook Lee

**Affiliations:** School of Computing and Augmented Intelligence, Arizona State University, Tempe, AZ 85281, United States; Biodesign Institute, Arizona State University, Tempe, AZ 85281, United States; School of Computing and Augmented Intelligence, Arizona State University, Tempe, AZ 85281, United States; Biodesign Institute, Arizona State University, Tempe, AZ 85281, United States; Google DeepMind, Mountain View, CA 94043, United States; School of Computing and Augmented Intelligence, Arizona State University, Tempe, AZ 85281, United States; Biodesign Institute, Arizona State University, Tempe, AZ 85281, United States

## Abstract

**Motivation:**

Reliable TCR-epitope binding prediction models are essential for development of adoptive T cell therapy and vaccine design. These models often struggle with false positives, which can be attributed to the limited data coverage in existing negative sample datasets. Common strategies for generating negative samples, such as pairing with background T cell receptors (TCRs) or shuffling within pairs, fail to account for model-specific vulnerabilities or biologically implausible sequences.

**Results:**

To address these challenges, we propose an iterative attack-and-defend framework that systematically identifies and mitigates weaknesses in TCR-epitope prediction models. During the attack phase, a reinforcement learning from AI feedback (RLAIF) framework is used to attack a prediction model by generating biologically implausible sequences that can easily deceive the model. During the defense phase, these identified false positives are incorporated into fine-tuning dataset, enhancing the model’s ability to detect false positives. A comprehensive adversarial negative dataset can be obtained by iteratively attacking and defending the model. This dataset can be directly used to improve model robustness, eliminating the need for users to conduct their own attack-and-defend cycles. We apply our framework to five existing binding prediction models, spanning diverse architectures and embedding strategies to show its efficacy. Experimental results show that our approach significantly improves these models’ ability to detect adversarial false positives. The combined dataset constructed from these experiments also provides a benchmarking tool to evaluate and refine prediction models. Our framework offers a new approach for improving model robustness in other biological tasks where negative sampling is inherently limited.

**Availability and implementation:**

The curated dataset and code are available at a public repository (https://github.com/Lee-CBG/BAP_Attack_n_Defend).

## 1 Introduction

T cells are a vital component of the adaptive immune system, responsible for identifying and eliminating infected or abnormal cells. Such a process is mediated through T cell receptors (TCRs), specialized proteins expressed on the surface of T cells. TCRs recognize specific antigens that are presented on the surface of cells by major histocompatibility complexes (MHCs). Together, the peptide-MHC (pMHC) complex forms a molecular interaction platform that enables T cells to distinguish between self and non-self antigens (La Gruta *et al.* 2018). A binding between a T cell receptor (TCR) and an epitope (a part of an antigen important for recognition) triggers an immune response, enabling immune systems to target and eradicate diseased or infected cells.

Accurate prediction of TCR-epitope binding is crucial for advancing understanding of immune responses (Schumacher *et al.* 2019). Important applications of such prediction models include immunological treatments and vaccine design (Sbai *et al.* 2001). Computational prediction of TCR-epitope binding can serve as a cost-effective pre-screening tool to complement expensive and time-consuming wet-lab experiments. However, the task is complicated by the need to generalize across a vast and diverse sequence space of both TCRs and epitopes (Lythe *et al.* 2016). The TCR repertoire of an individual is thought to be in the order of $10^{10}$ and V(D)J recombination and junctional diversity can generate up to $10^{15}$ unique sequences.

A major challenge in developing TCR-epitope binding affinity prediction (BAP) models is scarcity and limited coverage of high-quality training data. While experimentally validated binding pairs (positive samples) are available in public databases, such as IEDB (Vita *et al.* 2019), McPAS (Tickotsky *et al.* 2017), and VDJdb (Shugay *et al.* 2018), the lack of non-binding pairs necessitates the curation of negative samples. Typical curation strategies include shuffling TCRs between different epitopes (Springer *et al.* 2021, Cai *et al.* 2022, Jokinen *et al.* 2023) and pairing epitopes with TCRs drawn from healthy repertoires (Zhang *et al.* 2022, 2023a, Gao *et al.* 2023). However, there is no guarantee that the curated pairs are truly non-binding. Although experimentally validated non-binding pairs exist (Montemurro et al. 2021), the data are limited to a very small number of epitopes, insufficient for generalization across broader epitopes.

Moreover, artificially paired negative samples often fail to capture full biological complexities or diversities of true non-binding interactions, introducing biases that undermine model generalizability (Moris *et al.* 2021, Dens *et al.* 2023). Existing studies have been primarily focusing on improving model architectures (Springer *et al.* 2021, Weber *et al.* 2021, Zhang *et al.* 2022, Gao *et al.* 2023) or optimizing the ratios of positive to negative samples (Montemurro *et al.* 2021), rather than addressing the inherent limitations of the datasets.

We recently observed that existing BAP models can make obvious mistakes, falsely identifying biologically implausible TCRs as binding to epitopes. For example, sequences like AY or VTGDGNDDYFDLEQVMMFDGSGNDN are significantly shorter than typical TCRs or lack essential conserved motifs found in genuine ones, yet BAP models incorrectly predict these as binding TCRs. We observed these inherent vulnerabilities while training an epitope-specific TCR generation model (Hesslow *et al.* 2022) using reinforcement learning, where the BAP model served as the reward function. This revealed that BAP models can be easily misled by adversarial TCR sequences that maximize reward output but are biologically implausible. This is not limited to a certain BAP model; it is common across BAP models with various architectures and embedding techniques, while characteristics of the false positive sequences may vary. Although the issue can be partially mitigated by simple heuristics, such as filtering by sequence length or regular expressions, these errors ultimately expose a significant lack of diversity and coverage in the training data. We hypothesize that the issue stems from current negative training samples which rely on a narrow range of authentic TCR sequences and lack representation of biologically unrealistic sequences.

Our goal is to address the vulnerabilities in TCR-epitope prediction models and ultimately to enhance the models’ generalization by pinpointing negative data curation. We introduce an iterative attack-and-defend framework using a reinforcement learning from AI feedback (RLAIF) (Ouyang *et al.* 2022) in a novel way. RLAIF helps to reduce hallucinations, seem plausible but are factually incorrect outputs, in large language models (LLMs) by leveraging feedbacks from reward models (Ji *et al.* 2023). In our context, hallucinations refer to non-binding TCR sequences that mislead the reward model into classifying them as binding. We adapt it to deliberately produce hallucinations and identify false positives. These false positives are then utilized to iteratively refine the prediction model, thereby addressing its weaknesses and enhancing its resilience.

Our experiments are performed across diverse model architectures (Montemurro *et al.* 2021, Springer *et al.* 2021, Cai *et al.* 2022, Zhang *et al.* 2022, 2023a) and embedding strategies (Henikoff and Henikoff 1992, Zhang *et al.* 2023a). In Section 5.1, we highlight that all tested models are susceptible to generating false positives, not only biologically wrong or misleadingly similar to real sequences. In Section 5.2, we demonstrate that model-specific negative datasets generated through our framework significantly improve model robustness and performance. In Section 5.3, we show that each model achieve superior performance on both binding and non-binding pairs when trained with a combined negative dataset from multiple models, compared to relying solely on their own generated data.

Our work delivers several notable contributions. We introduce a novel approach by which TCR-epitope binding affinity predictors can commonly learn non-biological engagement rules. This method refines BAP boundaries, potentially mitigating reward hacking in reinforcement learning from human feedback (RLHF) when BAP is used as a reward model and enhancing TCR-epitope binding classification. Moreover, our framework can be applied in many other domains like antibodies and small molecules with the suite of authenticity metrics tailored to be more discerning about what is a false positive. In addition, we provide the curated adversarial negative dataset produced through our experiments so that it can be used by future researchers obviating the need for generating their own adversarial examples. This dataset can serve as a complementary addition to existing training data to improve model robustness and performance. The curated dataset and code are available at a public repository (https://github.com/Lee-CBG/BAP_Attack_n_Defend).

## 2 Related works

We provide an overview of recent advances in TCR-epitope binding prediction models, discuss existing strategies of negative sample generation, and explore applications of RLAIF in related contexts.

### 2.1 TCR-epitope binding prediction models

Improvement of TCR-epitope binding prediction models has primarily been through the exploration of different neural network architectures and embedding techniques. For example, netTCR-2.0 (Montemurro *et al.* 2021) and TCRconv (Jokinen *et al.* 2023) employ convolutional neural networks (CNNs) to learn local dependency between nearby amino acid residues, while models such as ERGO (Springer *et al.* 2021) and TCRpeg (Jiang and Li 2023) utilize recurrent neural networks (RNNs) to capture sequential dependency between residues. ATM-TCR (Cai *et al.* 2022) and TITAN (Weber *et al.* 2021) leverage the self-attention mechanism to learn contextual relationships between amino acid residues within TCR sequences or between TCRs and epitopes. Various embedding techniques of amino acid sequences have also been explored. Static embeddings often utilizing BLOSUM62 (Henikoff and Henikoff 1992), assigns a static vector that captures inherent properties of each amino acid. Context-aware embeddings, including catELMo (Zhang *et al.* 2023a), TCRpeg (Jiang and Li 2023) and TCRBert (Wu et al. 2024), employ deep learning models to learn dynamic representations that reflect the specific sequence context. Despite the substantial progress in model architectures and embeddings, dataset curation and properties has received little attention (Zhang *et al.* 2023b).

### 2.2 Negative sample generation

TCR-epitope binding prediction is often formulated as a binary classification task (Montemurro *et al.* 2021, Springer *et al.* 2021, Zhang *et al.* 2022, 2023a). Experimentally validated TCR-epitope pairs accessible from public databases, serve as positive samples for the development of computational models. However, due to the extreme scarcity of experimentally validated non-binding TCR-epitope pairs, researchers must curate their own negative samples. Two widely used methods for generating negative samples are: (i) Negative shuffling (Springer *et al.* 2021, Cai *et al.* 2022, Jokinen *et al.* 2023, Zhang *et al.* 2023a), which shuffles TCRs between different epitopes, assuming that a TCR specific to one epitope is unlikely to bind to another; and (ii) Healthy Repertoire Sampling (Gao *et al.* 2023, Zhang *et al.* 2022, 2023a), which samples TCRs from healthy repertoires under the assumption that these TCRs are not likely to bind to epitopes associated with diseases or pathogens. Despite their widespread use, these strategies do not guarantee that the generated TCR-epitope pairs are truly non-binding and are limited to known TCRs.

### 2.3 Reinforcement learning from AI feedback and hallucinations

RLHF has emerged as powerful techniques for fine-tuning large language models (Ouyang *et al.* 2022), particularly in mitigating undesired behaviors such as generating inaccurate or irrelevant content, commonly referred to as hallucinations. RLHF uses human-labeled feedback to guide generation models to produce outputs aligned with predefined preferences. However, the reliance on human feedback makes this approach expensive and time-consuming, limiting its scalability. As an alternative, RLAIF learns a reward model on human-labeled feedback and then uses it to guide generation models without requiring continuous human intervention. RLAIF is cost effective and time efficient, hence broadly used in real-world applications (Christiano *et al.* 2017, Zheng *et al.* 2018, Stiennon *et al.* 2020). However, one of its caveats is that the quality of generated results heavily depends on the accuracy and reliability of reward model’s feedback.

In TCR-epitope binding prediction, RLAIF remains underexplored. Most applications of RLAIF focus on reducing hallucinations to improve model reliability (Christiano *et al.* 2017, Zheng *et al.* 2018, Stiennon *et al.* 2020), but in this study, we take a novel approach by leveraging RLAIF to deliberately generate adversarial examples—sequences that maximize the reward model’s outputs while lacking biological plausibility. These adversarial sequences expose critical vulnerabilities in reward models (TCR-epitope binding prediction models) and provide valuable insights into improving their robustness.

## 3 Methods

**Figure 1.**
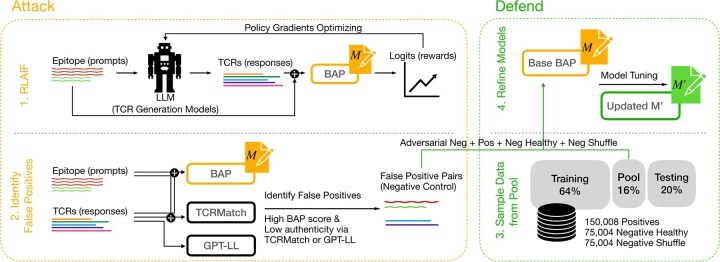
Overview of our iterative attack-and-defend framework. The methodology consists of two main phases: **Attack**. A supervised fine-tuned large language model (LLM) generates TCR sequences in response to epitope prompts. These sequences are evaluated by a BAP model (**M**), with proximal policy optimization (PPO) optimizing the generation process based on reward logits derived from **M**. Subsequently, auxiliary models (BAP, TCRMatch, GPT-LL) are used to identify false positives, sequences that score high on the BAP model but low scores from either TCRMatch or GPT likelihood-based evaluations. **Defend**. The identified false positives are combined with additional groups, including positive binding pairs, negative healthy pairs, and negative shuffled pairs, sampled from a reserved pool with desired ratios. The BAP model (Base **M**) is then fine-tuned on this curated dataset to enhance its robustness and performance. The updated model (**M’**) goes to next iterations until no significant amount of false positives can be found.

Our attack-and-defend framework systematically identifies and addresses vulnerabilities in TCR-epitope binding prediction models (Fig. 1). The framework consists of iterating two main phases executed alternately: attack and defense. The attack phase seeks to uncover negative samples that BAP models falsely predict as binding. The defense phase refines BAP models by further fine-tuning with the newly identified negative pairs, in addition to existing positive and negative pairs. Through iteration, the models improve generalization capabilities, and the process stops when attacks no longer generate significant false positives.

### 3.1 Attack

The attack phase consists of two subphases: generating TCR sequences via an RLAIF framework (Section 3.1.1) and identifying sequences where prediction models fail (Section 3.1.2). We also provide details on the TCR sequence generation model that serves as a policy in the RLAIF framework (Section 3.1.3), and the binding affinity prediction model, targeted for attack (Section 3.1.4).

#### 3.1.1 RLAIF training with BAP models

To attack prediction models, we incorporated a fine-tuned TCR sequence generation model into a RLAIF framework. In this setup, the generation model (detailed in Section 3.1.3) acts as the policy $\pi_{\theta }$, while the prediction models being attacked serve as reward models *R*, providing feedback to the RL framework. The reward signal, r(t), is derived from the logit outputs of the reward model before applying sigmoid activation functions. This choice ensures optimization is performed over a continuous and unbounded space, which is more gradient-friendly compared to discrete probabilities. Mathematically, the reward signal is expressed as:


r(t)=logit(M(t,e)),

where *t* represents the generated TCR sequence, *e* is the target epitope, and M(t,e) is the raw logit output of the prediction model for the *t*-*e* pair.

The objective of the attack phase is to maximize the expected reward for sequences sampled from the policy $\pi_{\theta }$. This is formalized as:


$$J(\theta )=E_{t∼\pi_{\theta }}[r(t)].$$

A successful attack is characterized by the generation of sequences that achieve high reward scores (indicating strong likelihood of binding) while being biologically implausible or unrealistic. Examples of such unrealistic sequences include: repeating amino acid residues (e.g. EEEE.) or excessively short or nonsensical sequences (e.g. AY).

#### 3.1.2 Identification of false positives

Sequences are defined as false positives if they achieve high BAP reward scores in the RLAIF framework but score low on authenticity metrics. Two methods [TCRMatch (Chronister et al. 2021) and GPT-LL (Zhang *et al.* 2025)] are used to evaluate the authenticity of generated sequences.

**TCRMatch** is a k-mer based sequence comparison method that evaluates the sequence similarity of TCR sequences against a reference database of known sequences. While it does not directly predict binding affinity to an epitope, a high similarity score to a known set of its binding TCRs implies that the query sequence might possess binding characteristics similar to TCRs known to recognize the epitope. The similarity score is computed by breaking TCRs into overlapping k-mers and comparing them to a database of known binding TCRs. Sequences with low similarity scores are flagged as potential non-binding, as they deviate significantly from known biological patterns. Based on our previous evaluation experiments (Zhang *et al.* 2025), we found that TCRMatch is particularly effective at distinguishing sequence authenticity. Therefore, we include it as one of the measures for evaluating the authenticity of TCR sequences.

**GPT-LL** is a transformer-based large language model trained on genuine TCR sequences to learn their inherent patterns. It outputs log-likelihood score of a TCR sequence representing its authenticity. GPT-LL utilizes the same model architecture as our TCR generation model (Section 3.1.3). It is fine-tuned, upon a pre-trained protein sequence model (Hesslow *et al.* 2022), exclusively on TCR sequences without conditioning on epitopes (see Section 4.3 for dataset used).

For a given TCR sequence *t* composed of *L* amino acid residues $\left(a_{1},a_{2},\ldots ,a_{L}\right)$, the model computes total log-likelihoods by summing the conditional probabilities of each amino acid in a given TCR sequence $P(a_{i}�|a_{<i})$. The overall authenticity of TCR *t* is then quantified as the sum of the log-likelihoods of its residues:


$$S_{\text{GPT-LL}}(t)=\sum_{i=1}^{L}\;\text{log}\;P(a_{i}|a_{<i}),$$

where $a_{<i}=(a_{1},a_{2},\ldots ,a_{i-1})$ represents preceding residues of the *i*-th residue. A higher score indicates a greater likelihood that a TCR sequence originates from the authentic TCR distribution, whereas adversarial or unrealistic sequences typically yield lower scores.

#### 3.1.3 TCR generation model

The TCR generation model is used to produce TCR sequences during attack via RLAIF. We fine-tuned a large protein language model using known binding TCR-epitope pairs (Hesslow *et al.* 2022). Let the dataset of binding pairs be represented as ${\left\{(t_{i},e_{i})\right\}}_{i=1}^{N}$, where $t_{i}$ is the TCR sequence and $e_{i}$ is the corresponding epitope. The fine-tuning objective is to maximize the likelihood of the observed data:


$$L(\theta )=\sum_{i=1}^{N}\;\text{log}\;\pi_{\theta }(t_{i}|e_{i}),$$

where $\pi_{\theta }$ is the generation model parameterized by θ.

This supervised fine-tuning ensures the generation model learns meaningful sequence patterns and context-specific relationships between epitope and TCR sequences. By conditioning on epitopes, the model generates sequences that are biologically plausible and consistent with observed data. The resulting fine-tuned model serves as the reference policy $\pi_{\text{ref}}$ in the RLAIF framework.

#### 3.1.4 Base BAP models

To construct robust negative samples and evaluate model robustness, we included a diverse set of prediction models spanning a range of neural network architectures and amino acid embedding methods (Table 1). Each model represents a different approach to TCR-epitope binding prediction, incorporating distinct embedding strategies. For a fair comparison, all base BAP models were trained and tested on the same TCR-epitope pairs, maintaining a consistent ratio of positive to negative shuffle to negative healthy samples as 2:1:1. Each model was trained using cross-entropy loss with a learning rate of 0.001 for 200 epochs, and the best-performing checkpoint was saved for evaluation.

**Table 1.** List of Base BAP models covered in our study.

Model name	Architecture	Embeddings	Original negative set
ATM-TCR (Cai *et al.* 2022)	Self-attention	BLOSUM	Negative shuffle pairs
ERGO-LSTM (Springer *et al.* 2021)	LSTM	BLOSUM	Negative shuffle pairs
PiTE (Zhang *et al.* 2022)	Self-attention	catELMo	Negative healthy pairs
catELMo MLP (Zhang *et al.* 2023a)	MLP	catELMo	Negative healthy pairs
netTCR-2.0 (Montemurro *et al.* 2021)	CNN	BLOSUM	Validated negative pairs

### 3.2 Defend

The defense phase consists of two subphases: (i) curating a fine-tuning dataset that includes both positive and negative pairs, and (ii) fine-tuning the base BAP models on the curated dataset to improve robustness.

#### 3.2.1 Sample data

We create a balanced fine-tuning dataset by combining the adversarial negative samples and those sampled from a reserved pool including positive binding pairs, negative healthy pairs, and negative shuffled pairs (see Section 4). Positive binding pairs are TCR-epitope pairs that have been experimentally validated to bind (binding = 1). Negative healthy pairs are generated by pairing TCRs sampled from healthy repertoires with epitopes (binding = 0). Negative shuffled pairs are created by shuffling known TCR-epitope pairs (binding = 0). The false positives, referred to as adversarial negative pairs, consist of false positives identified during the attack phase.

The sampling is performed with a certain ratio of the groups to maintain balance of contributions of the adversarial examples with the other groups, thereby enhancing the model’s generalization ability while preventing overfitting to any single group. When defending different models, the ratios are adjusted to achieve optimal performance.

#### 3.2.2 Refine BAP models

We enhance the robustness of prediction models by improving their ability to distinguish authentic binding pairs while reducing the false positive rate. Base BAP models (*M*) are fine-tuned on the curated dataset with a reduced learning rate of $10^{-5}$, which is 100 times lower than the initial training for the base BAP models. The curated fine-tuning dataset includes TCR-epitope pairs sampled from all the groups: positive, negative healthy, negative shuffle and adversarial negatives (Section 3.2.1). Each model is fine-tuned for 200 epochs, with the best-performing checkpoint ($M^{\prime }$) saved based on validation loss. In each iteration, the best checkpoint is reserved for the next iteration’s base model.

## 4 Data

Two distinct types of datasets were prepared: (i) labeled TCR-epitope pairs for training and evaluating prediction models and generation models, and (ii) unlabeled TCR sequences sampled from healthy repertoires, used to evaluate sequence authenticity. This study utilized only sequence data, specifically the beta chain CDR3 region, which we refer to as the TCR sequence unless otherwise specified.

### 4.1 TCR-epitope pairs

#### 4.1.1 Positive pairs

We collected TCR-epitope binding pairs from three public databases: VDJdb (Shugay *et al.* 2018), IEDB (Vita *et al.* 2019), and McPAS (Tickotsky *et al.* 2017). We preprocessed the samples as described in ATM-TCR (Cai *et al.* 2022), resulting in 150 008 positive TCR-epitope pairs. We also generated 150 008 negative pairs as experimentally validated non-binding pairs are scarce.

#### 4.1.2 Negative shuffle pairs

Half of the negative samples were curated by shuffling the positive pairs. The assumption is that TCRs binding to a diseased epitope are unlikely to bind to another diseased epitope. These shuffled pairs are referred to as negative shuffle samples.

#### 4.1.3 Negative healthy pairs

The remaining half of the negative samples were generated by pairing epitopes with TCR sequences randomly sampled from healthy repertoires. The underlying hypothesis is that TCRs derived from healthy individuals are unlikely to interact with diseased epitopes. These generated pairs are referred to as negative healthy samples.

### 4.2 Usage

The labeled TCR-epitope pairs were used for two primary models. The first usage is on training and evaluating the BAP models. Both positive and negative pairs were used to train and evaluate the prediction models. The dataset was split as follows: 64% for training the base prediction models, 16% reserved as a pool for iterative defending (during the attack-and-defend framework), and 20% for testing. The second usage is on supervised fine-tuning of the TCR generation model. Only positive pairs were used for fine-tuning the TCR generation model. The dataset was split into 80% for training and 20% for testing.

### 4.3 Unlabeled TCRs

We also collected 4M TCR sequences from healthy repertoires (Nolan *et al.* 2025). These sequences do not have associated epitope binding information and were filtered by the criteria described in catELMo (Zhang *et al.* 2023a). They were used to develop GPT-LL model, which learns authentic TCR sequences and hence detects biologically implausible sequences. We used 64% of the sequences for training, 16% for validation and 20% for testing.

## 5 Results

Our framework iteratively refines BAP models, reducing false positive rates. We first show that all tested BAP models are susceptible to adversely generated sequences that are not only biologically implausible but also deceptively similar to authentic sequences. Next, we demonstrate that iterative attack-and-defend strategies effectively improve the robustness of BAP models by reducing false positives. Finally, we offer a curated adversarial negative dataset, derived from the multiple models, which can serve as a valuable resource for other researchers.

### 5.1 BAP attack is a universal problem

**Figure 2.**
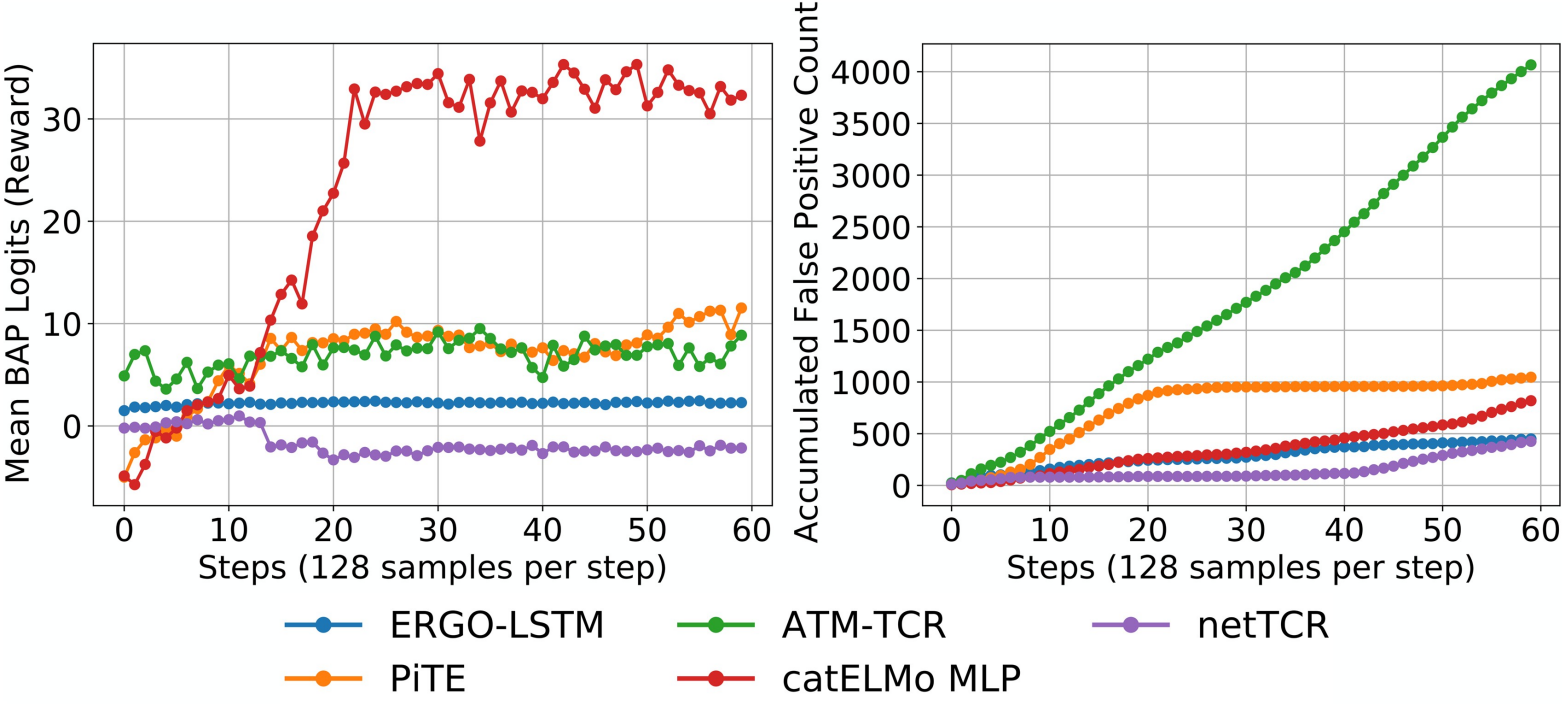
Evaluation of TCR sequence generations across BAP models. The mean BAP logits (reward values) for generated sequences consistently increase over the generation steps for all prediction models (Left). The accumulated false positive counts also increase over steps (Right).

We successfully attacked all five tested models, each employing distinct neural network architectures and amino acid embedding techniques (Table 1). All models tend to assign high binding scores to the adversarially generated TCRs, as reflected in their logit outputs. We tracked the mean BAP logit scores of all generated sequences over proximal policy optimization (PPO) steps (Fig. 2, left). Most sequences have scores above 0, corresponding to predicted binding probabilities greater than 0.5. This suggests that BAP models confidently predict many generated TCR sequences as binders. However, many of these high-scoring sequences are not biologically meaningful and they are identified by TCRMatch and GPT-LL as false positives. For most models, the number of false positives accumulates across PPO steps (Fig. 2, right), reveals persistent vulnerabilities that BAP attacks can exploit.

**Figure 3.**
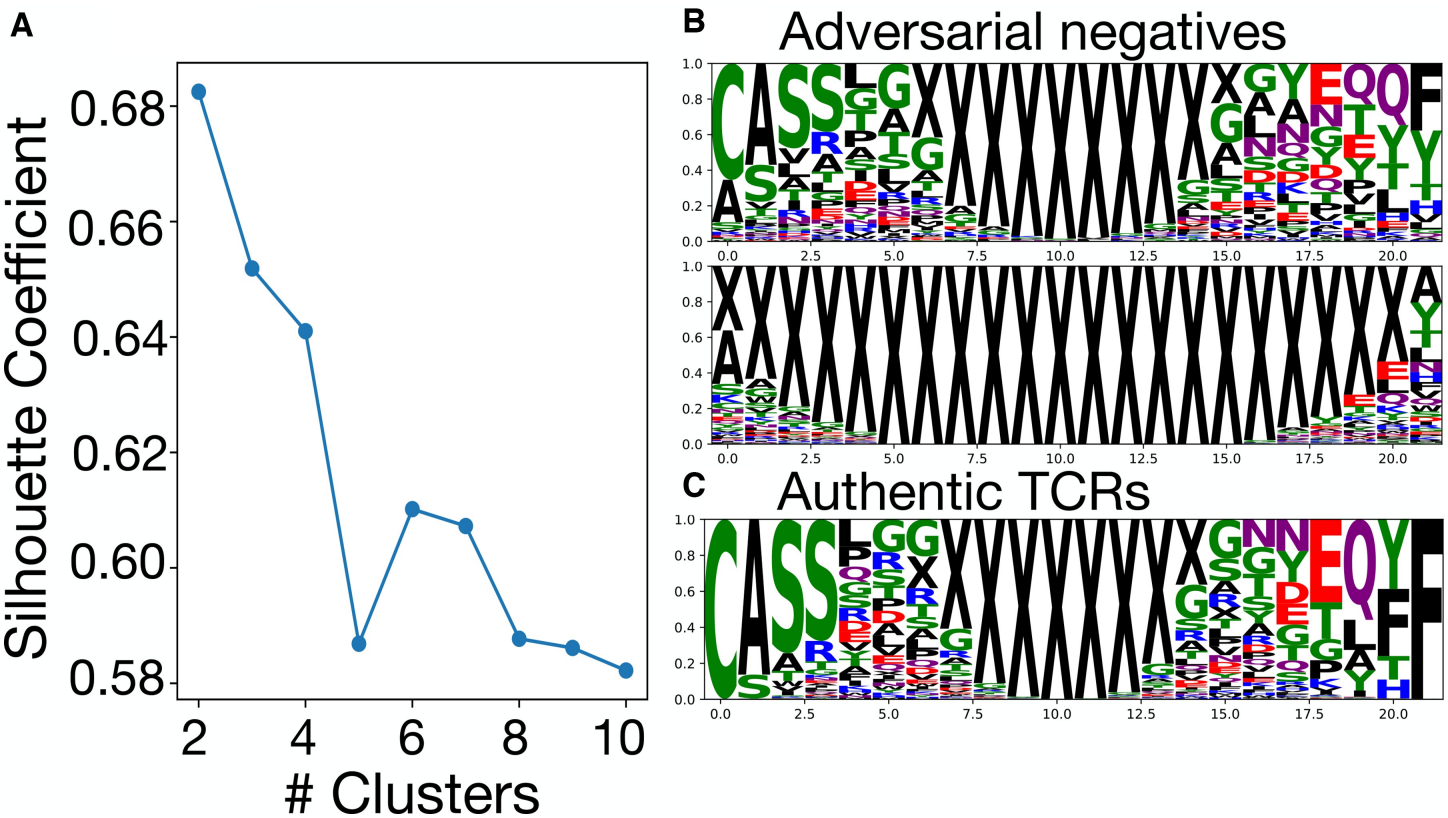
Clustering and motif analysis of sequences generated from attacking catELMo MLP model. (A) Silhouette scores for clustering adversarially generated sequences. (B) SeqLogo visualizations of the two identified clusters. (C) SeqLogo visualizations of authentic TCRs.

To better understand the patterns of the false positives, we clustered the sequences using TCRdist (Dash *et al.* 2017), pairwise distance metric based on similarity-weighted hamming distances. The optimal number of clusters was determined using silhouette scores (Rousseeuw 1987). We visualized sequence motifs of the resulting clusters using SeqLogo diagrams for catELMo MLP (Fig. 3) and compared them against that of authentic TCR sequences. The motifs from the false positive clusters exhibit both similarities and dissimilarities when compared to authentic sequences. Some retain conserved motifs, particularly at the beginning of the CDR3 region, making them resemble natural TCRs (Fig. 3B, top). Such outcome is expected, as prediction models rely on learned sequence patterns to determine binding probability. Other generated sequences exhibit clearly distinct motifs compared to authentic sequences (Fig. 3B, bottom), indicating that our attack-and-defend framework exposes a spectrum of model vulnerabilities—ranging from sequences that closely resemble natural TCRs to unrealistic sequences yet still receive high binding scores. Visualizations for other tested prediction models show a similar trend (Fig. A1, available as supplementary data at *Bioinformatics* online), with adversarial negatives spanning from natural-like to clearly non-biological patterns.

### 5.2 Fine-tuning improves robustness against generated false positives

Fine-tuning TCR-epitope prediction models with the refined negative set significantly enhances model robustness. During the defense phase, adversarial negative sequences were supplemented with three additional groups—positive binding pairs, negative shuffle pairs, and negative healthy pairs—drawn from a reserved data pool. To ensure balanced learning, we systematically optimized the ratios of these four groups for each model.

**Figure 4.**
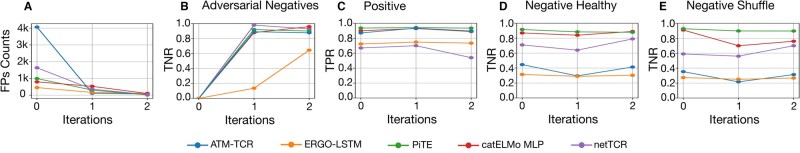
False positive counts and BAP performance per data group over attack-and-defend iterations. (A) The number of identified false positives (FPs) decreases significantly as the iterations progress, with negligible FPs observed by the third iteration. (B) Significant performance boost on adversarial negative dataset. (C–E) Performance for positive, negative shuffle, and negative healthy groups remain stable. True positive rate (TPR) or true negative rate (TNR) were reported for positive and negative group, respectively, as each group contains only a single label class.

Our results show that as the iterations progress, the overall number of false positives decreases significantly (Fig. 4A). For all models, we stopped the attacks by the third iteration, as the number of false positives became negligible. To show how attack-and-defend iterations affect BAP model performance on different data types, we report results per data group. After the first round fine-tuning, prediction models showed marked improvement in mitigating their initial vulnerabilities to false positives, as evidenced by significant increases in performance scores on the adversarial negative data (Fig. 4B). Furthermore, most accuracy scores for the other three groups (positive, negative shuffle, and negative healthy) remained stable compared to their initial performances (Fig. 4C–E). Overall, we show that our attack-and-defend framework improves model robustness against adversarially generated sequences. Such improvement also suggests that our curated adversarial negative dataset offers broader data coverage compared to previous datasets.

### 5.3 Our adversarial negative dataset improve BAP robustness without additional attacks

The combined adversarial negative dataset serves as a valuable resource for enhancing the data coverage and improving the robustness of TCR-epitope prediction models. In total, we collected 9694 adversarial negative pairs generated through three iterations of our attack-and-defend framework, applied across five distinct prediction models.

**Figure 5.**
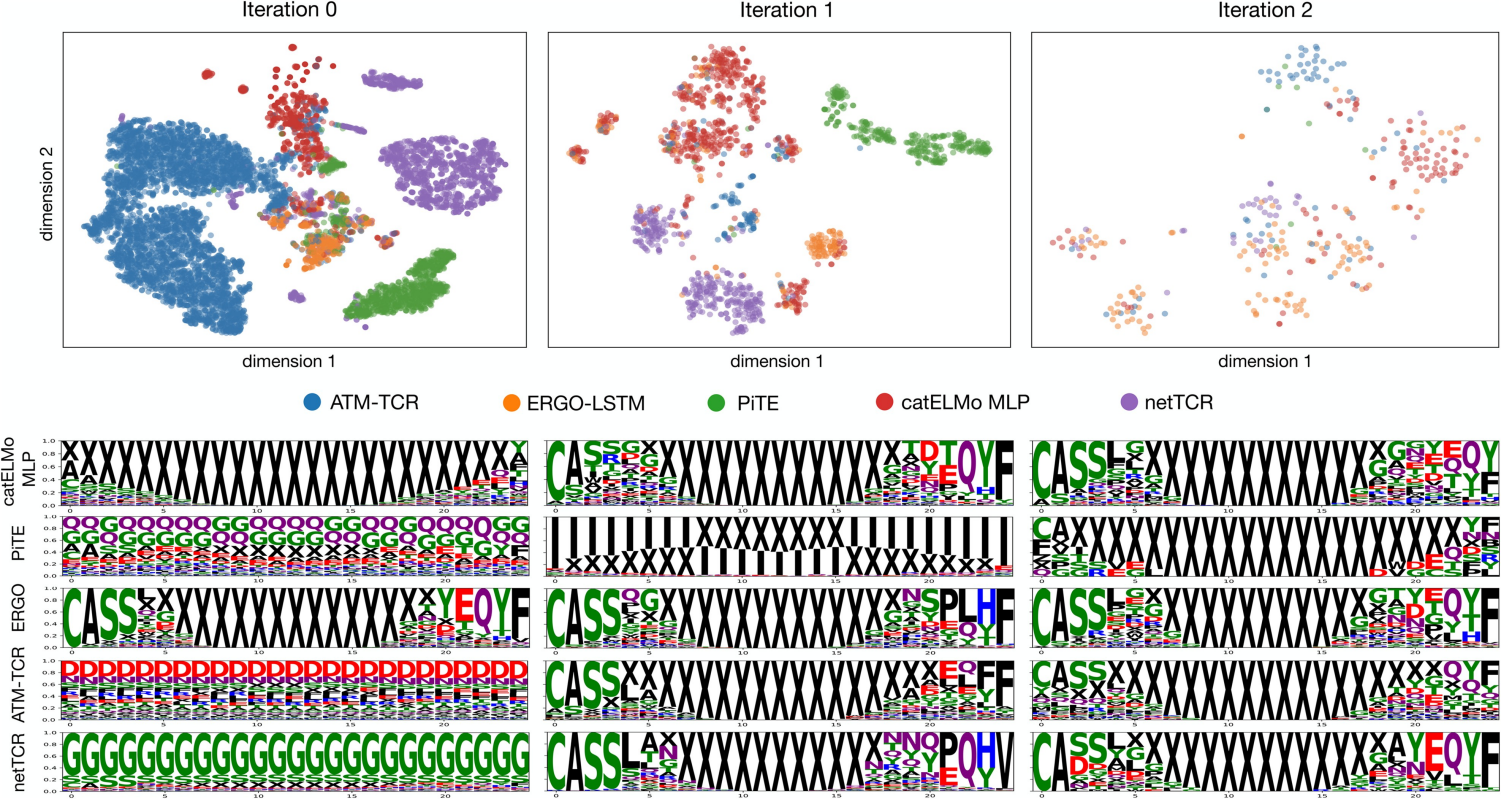
Visualization of TCR embeddings from different source models across attack iterations. (Top) t-SNE illustrates the distribution of TCR embeddings for five source models across three attack iterations. Each data point represents a TCR embedding, and colors indicate the source model. The sample counts for each source model are displayed in the legend. It highlights the evolving diversity and clustering of adversarial TCR embeddings over successive attack iterations. (Bottom) Sequence motifs from false positive groups, visualized through SeqLogo diagrams, showed increased resemblance to authentic sequences over iterations, particularly in the initial and final k-mers.

Our iterative framework refines negative examples over multiple attack-and-defend cycles, producing sequences that are both harder to distinguish from genuine binders and more model-agnostic. We visualized the identified adversarial negative dataset using t-SNE plots and SeqLogo visualizations (Fig. 5). First, all adversarial negative sequences were embedded using catELMo embeddings and subsequently reduced to two dimensions for visualization. The t-SNE plot reveals that adversarial negatives from each prediction model form distinct clusters in the first two iterations, indicating clear differences between the vulnerabilities of the models. By the third iteration, these distinctions become less clear, with data points appearing more mixed. This trend is likely due to two factors: (i) fewer adversarial negatives were identified in later iterations, and (ii) as model vulnerabilities were progressively addressed, the adversarial negative became more similar across models. Similar patterns can be observed in the SeqLogo plots, where false positives from the first two iterations are easy to distinguish but, in later iterations, appear more similar to each other and to real sequences.

Of these false positives, the first two iterations primarily reveal “easy” false positives, such as sequences that are unrealistically short or consist of repeating amino acid residues. These vulnerabilities can often be addressed through simple heuristic filtering. However, after defending the models using false positives from these earlier iterations, the models progressively discover “harder” false positives—sequences that fail the models but are more nuanced, exhibiting greater similarity to both real sequences and to each other. This shows the effectiveness of our attack-and-defend framework in exposing deeper, harder-to-detect vulnerabilities in TCR-epitope prediction models.

To demonstrate the utility of the curated adversarial negative dataset, we directly fine-tuned base prediction models using these samples, paired with three additional groups (positive binding, negative shuffle, and negative healthy). Compared to base models trained solely on the three groups, the fine-tuned models showed improved accuracy across all adversarial negative data.

Since the adversarial negative dataset includes many unrealistic sequences that can be trivially filtered, we applied simple heuristics—based on TCR length and repetitive patterns—to the base models. This serves a more meaningful baseline by removing obviously implausible negatives. As shown in Table 2, even with this filter in place, base models still misclassified around 20% of the adversarial negative sequences. However, all fine-tuned models reduced this error rate to below 10%, with several models dropping to under 5%, except ATM-TCR (still reduced slightly). This reflects a non-trivial improvement in robustness beyond what heuristics alone can provide. The gains also extended beyond adversarial negatives: the fine-tuned models achieved better or comparable performance to the base models (with additional heuristic or authenticity filtering) on the original three test groups—positive, negative shuffle, and negative healthy. This is also observed in the AUCs across these three groups (Table A1, available as supplementary data at *Bioinformatics* online). Even when replacing heuristic filters with authenticity metrics like GPT-LL and TCRMatch for the baseline, our approach still showed a significant improvement (Table A1, available as supplementary data at *Bioinformatics* online). This indicates that adversarially generated examples through our framework help refine the model’s decision boundary in a way that improves the overall classification performance. It is likely because some of the false positives are similar to authentic TCR sequences. Fine-tuning on the aggregated false positives may contribute to noticeable refinement of each model’s decision boundaries, particularly in areas where they share common vulnerabilities.

**Table 2.** BAP performance (averaged across three runs) after fine-tuning on the combined dataset.^a^

	Adversarial negatives			Positive			Negative healthy			Negative shuffle		
Model	Base	Base w/Heuristics	After FT (Ours)	Base	Base w/Heuristics	After FT (Ours)	Base	Base w/Heuristics	After FT (Ours)	Base	Base w/Heuristics	After FT (Ours)
ATM-TCR (Cai *et al.* 2022)	23.21	79.29	79.61 (0.59)	79.08	79.08	79.09 (0.33)	57.11	57.11	54.44 (1.01)	42.42	42.42	41.71 (1.13)
ERGO-LSTM (Springer *et al.* 2021)	30.88	81.56	91.66 (0.22)	67.97	67.88	68.67 (6.93)	37.21	37.25	58.86 (2.41)	33.69	33.85	46.65 (2.63)
PiTE (Zhang *et al.* 2022)	17.61	78.61	99.07 (1.08)	95.48	95.48	93.40 (2.69)	83.73	83.73	85.46 (2.41)	81.55	81.55	83.33 (1.64)
catELMo MLP (Zhang *et al.* 2023a)	11.67	85.49	99.03 (0.88)	90.70	90.60	93.39 (1.08)	78.31	78.31	80.71 (1.54)	86.22	86.26	96.67 (0.22)
netTCR-2.0 (Montemurro *et al.* 2021)	10.10	79.84	96.96 (0.20)	58.67	58.57	56.34 (0.42)	77.29	77.29	77.59 (1.68)	44.25	44.46	50.34 (1.27)

a This combined dataset includes all models’ adversarial negative pairs augmented by our attack-and-defend framework, and positive binding pairs, negative healthy pairs and negative shuffled pairs sampled from a reserved pool. For each data group, we report the either TPR or TNR of: (i) the base model, (ii) the base model with heuristic (length in the range 5–30 and repetition ≤6) filtering for authenticity, and (iii) the fine-tuned model (ours). Since each model was fine-tuned over multiple runs on the same base model, standard deviation is only reported (in parentheses) for the fine-tuned results. Highest scores within each data group are underlined.

## 6 Conclusion

We identified and addressed critical limitations of existing TCR-epitope prediction models focusing on negative data curation. Through our iterative attack-and-defend framework, we systematically uncovered model vulnerabilities to biologically implausible sequences and leveraged them to construct a comprehensive adversarial negative dataset. The refined dataset significantly improved model robustness and reduced false positive rates, demonstrating its effectiveness across a diverse range of prediction models.

Our experimental results demonstrate potential benefits of enhancing BAP models for both binding affinity prediction and their use as reward functions in TCR generation tasks. When used in RLAIF-based TCR sequence generation, our framework enables the training of more robust BAP models, preventing them from guiding generation toward biologically implausible sequences. Importantly, the fine-tuned models improve performance not only on adversarial negatives but also on realistic pairs (positive, negative-healthy, and negative-shuffle) as shown in Table 2, ultimately benefiting downstream binding affinity prediction.

It is noted that the degree of improvement varied across models. Such variation may stem from differences in model architectures or embedding strategies, which affect how sequence patterns are learned and how decision boundaries are formed. Consequently, RLAIF targets model-specific boundaries and iteratively generates different adversarial sequences across models, which may lead to varied training effectiveness due to their unique distributions. Future work should systematically characterize these differences to optimize attack-and-defend strategies for specific architectures. This may help to determine an optimal ratio of combining model-specific curated negative sets rather than a simple aggregation. Extending our framework to paired-chain TCR models is another promising future direction, especially as paired-chain repertoires and language models become more available (Raybould *et al.* 2024).

Conceptually, our framework shares similarities with generative adversarial networks (GANs) (Goodfellow *et al.* 2014), where a generator produces candidates to challenge a discriminator. In our framework, the TCR generation model (as a generator) generates adversarial TCR sequences to probe a BAP model (analogous to a discriminator). However, unlike GANs’ simultaneous min-max training, we alternate the attack and defense phases as separate steps. While GANs primarily focus on optimizing the generator to produce samples to deceive a discriminator, our framework focuses on refining BAP models by crafting negative samples that reveal specific vulnerabilities. Biological plausibility is not part of the generation objective but is instead used later as a filtering criterion during the defense phase.

While we focused on a representative set of architectures and embedding techniques, it is infeasible to evaluate every existing TCR-epitope prediction model. Other datasets, such as MIRA (Hudson *et al.* 2023), can also be integrated to extend our framework to a wider range of TCR-epitope data. Our attack-and-defend framework is adaptable and can be applied to any predictive model users wish to evaluate. This flexibility ensures that our framework can serve as a valuable tool for researchers aiming to enhance the reliability of their own models. More broadly, the framework can be extended to other biological problems where negative sampling remains an open challenge (Fuller *et al.* 2013).

## Supplementary Material

btaf224_Supplementary_Data
