# Supplementary material for: Iterative attack-and-defend framework for improving TCR-epitope binding prediction models
Source: Bioinformatics. 2025 Jul 15;41(Suppl 1):i429–38. doi: 10.1093/bioinformatics/btaf224 (PMC12261473; doi:10.1093/bioinformatics/btaf224)
Supplement: btaf224_Supplementary_Data [file btaf224_supplementary_data.pdf]

A1 Appendix

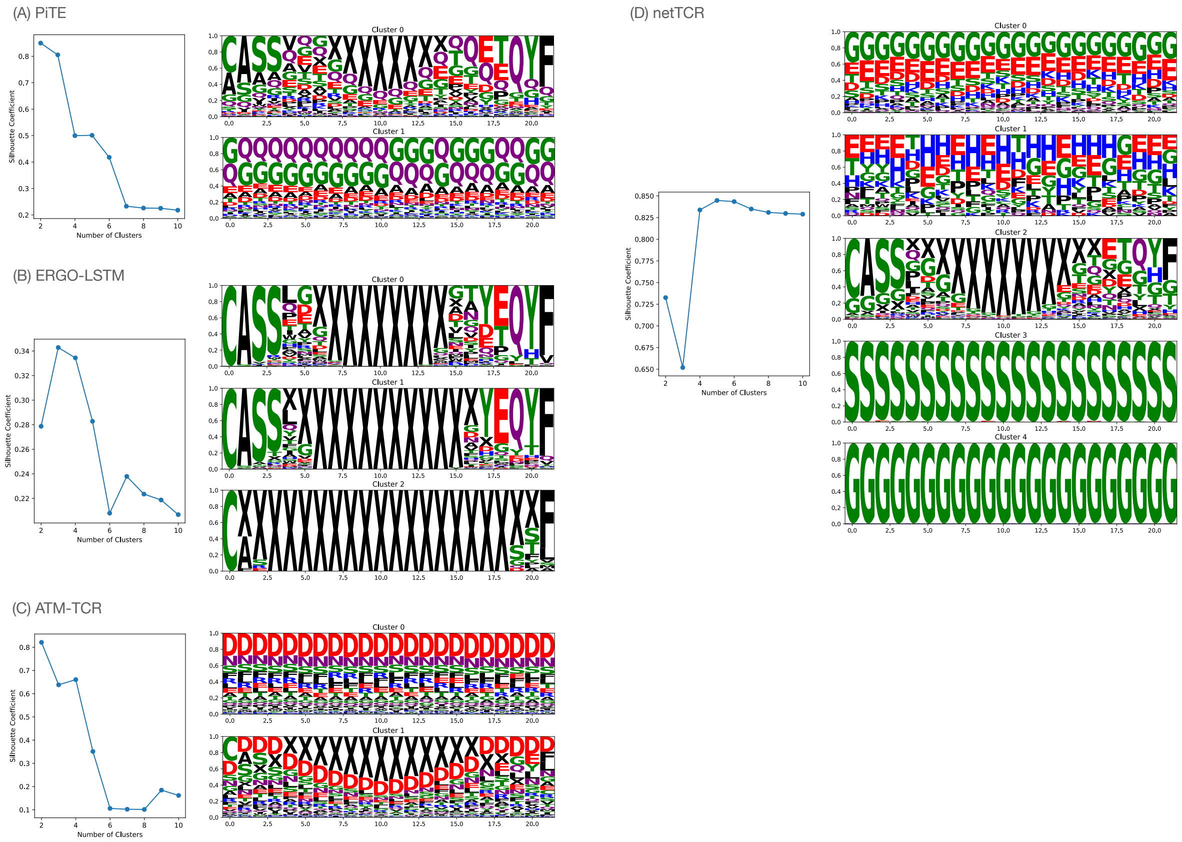

Figure A1: Clustering and motif analysis of sequences generated from attacking different prediction models. Silhouette scores for clustering adversarially generated sequences (Left) and SeqLogo visualizations of the identified clusters (Right) for (A) PiTE, (B) ERGO-LSTM, (C) ATM-TCR, and (D) netTCR.

Table A1: AUC scores (averaged over three runs) for each model across different data groups (positive, negative healthy, and negative shuffle) under various evaluation settings. We report AUCs for: (1) the base model, (2) the base model with heuristic filtering (based on sequence length and repetition), (3) the base model filtered by authenticity metrics (GPT-LL and TCRMatch), and (4) the fine-tuned model (ours). Since each model was fine-tuned over three runs on the same base model, standard deviation is only reported (in parentheses) for our fine-tuned results. Highest scores within each model are underlined.

**Model**	**Base**	**Base w/ Heuristics**	**Base w/ Authenticity**	**After FT (Ours)**
ATM-TCR	72.75	72.75	64.99	72.27 (0.25)
ERGO-LSTM	52.27	52.27	52.31	62.83 (1.88)
PiTE	96.44	96.44	79.45	96.38 (0.12)
catELMo MLP	94.53	94.53	78.78	97.88 (0.13)
netTCR-2.0	63.37	63.37	59.93	63.56 (0.05)
